# Cardiovascular risk factors and development of nomograms in an Italian cohort of patients with suspected coronary artery disease undergoing SPECT or PET stress myocardial perfusion imaging

**DOI:** 10.3389/fnume.2024.1232135

**Published:** 2024-02-14

**Authors:** Rosario Megna, Mario Petretta, Carmela Nappi, Roberta Assante, Emilia Zampella, Valeria Gaudieri, Teresa Mannarino, Adriana D’Antonio, Roberta Green, Valeria Cantoni, Mariarosaria Panico, Wanda Acampa, Alberto Cuocolo

**Affiliations:** ^1^ Institute of Biostructure and Bioimaging, National Council of Research, Naples, Italy; ^2^ IRCCS Synlab SDN, Naples, Italy; ^3^ Department of Advanced Biomedical Sciences, University Federico II, Naples, Italy

**Keywords:** cardiovascular risk factors, coronary artery disease, SPECT, PET, myocardial perfusion imaging, nomogram, pretest, receiver operating characteristic

## Abstract

**Introduction:**

Single-photon emission computed tomography (SPECT) and positron emission tomography (PET) are non-invasive nuclear medicine techniques that can identify areas of abnormal myocardial perfusion. We assessed the prevalence of cardiovascular risk factors in patients with suspected coronary artery disease (CAD) undergoing SPECT or PET stress myocardial perfusion imaging (MPI). Based on significant risk factors associated with an abnormal MPI, we developed a nomogram for each cohort as a pretest that would be helpful in decision-making for clinicians.

**Methods:**

A total of 6,854 patients with suspected CAD who underwent stress myocardial perfusion imaging by SPECT or PET/CT was studied. As part of the baseline examination, clinical teams collected information on traditional cardiovascular risk factors: age, gender, body mass index, angina, dyspnea, diabetes, hypertension, hyperlipidemia, family history of CAD, and smoking.

**Results:**

The prevalence of cardiovascular risk factors was different in the two cohorts of patients undergoing SPECT (*n* = 4,397) or PET (*n* = 2,457) myocardial perfusion imaging. A statistical significance was observed in both cohorts for age, gender, and diabetes. At multivariable analysis, only age and male gender were significant covariates in both cohorts. The risk of abnormal myocardial perfusion imaging related to age was greater in patients undergoing PET (odds ratio 4% vs. 1% per year). In contrast, male gender odds ratio was slightly higher for SPECT compared to PET (2.52 vs. 2.06). In the SPECT cohort, smoking increased the risk of abnormal perfusion of 24%. Among patients undergoing PET, diabetes and hypertension increased the risk of abnormal perfusion by 63% and 37%, respectively. For each cohort, we obtained a nomogram by significant risk factors at multivariable logistic regression. The area under the receiver operating characteristic curve associated with the nomogram was 0.67 for SPECT and 0.73 for the PET model.

**Conclusions:**

Patients with suspected CAD belonging to two different cohorts undergoing SPECT or PET stress myocardial perfusion imaging can have different cardiovascular risk factors associated with a higher risk of an abnormal MPI study. As crude variables, age, gender, and diabetes were significant for both cohorts. Net of the effect of other covariates, age and gender were the only risk factors in common between the two cohorts. Furthermore, smoking and type of stress test were significant for the SPECT cohort, where as diabetes and hypertension were significant for the PET cohort. Nomograms obtained by significant risk factors for the two cohorts can be used by clinicians to evaluate the risk of an abnormal study.

## Introduction

Single-photon emission computed tomography (SPECT) and positron emission tomography (PET) are non-invasive nuclear medicine techniques that can identify areas of abnormal myocardial perfusion (1–3). SPECT is based on gamma-emitting radionuclides, delivered to the patient through injection into the bloodstream. Gamma radiations emitted from the patient's body area under study are measured directly as counts from a rotating gamma camera, and images are obtained by reconstruction algorithms. PET, unlike SPECT, is based on positron-emitting radioisotopes. A positron emitted from a radionuclide travels a very short distance, losing its kinetic energy. At rest, it typically remains a time of a few hundred picoseconds before annihilating with an electron, and the masses of the two particles are converted into two 511 keV photons traveling in opposite directions. By the difference in arrival times of the photons to detectors (time of flight), it is possible to compute their starting point, which is where the annihilation occurred. PET images are obtained by counts and reconstruction algorithms (4). For the diagnostic and prognostic work-up of patients with suspected or known coronary artery disease (CAD), SPECT and PET cardiac imaging are usually performed at rest and after stress testing (2–6).

In the last few decades, large-scale implementation of preventive measures and development of new diagnostic, as well as therapeutic, approaches have reduced cardiovascular morbidity and mortality (7, 8). Epidemiologic data also show that improved control of cardiovascular risk factors has resulted in a temporal decrement in the incidence and severity of CAD and its related mortality (9–12). However, current guidelines on cardiovascular disease prevention in clinical practice concentrate principally on traditional risk factors (13). The purpose of this study was to assess the prevalence of cardiovascular risk factors in patients with suspected CAD undergoing SPECT or PET stress myocardial perfusion imaging (MPI). Based on significant risk factors associated with an abnormal myocardial perfusion, we developed a nomogram for each cohort. This tool could be helpful in decision-making for clinicians.

## Methods

### Study population

For this study, we included 6,854 patients of ages 18 years or older with suspected CAD who underwent myocardial perfusion imaging by SPECT (*n* = 4,397) or PET/CT (*n* = 2,457) from January 2012 to December 2022, as part of their diagnostic program according to the recommendations of the European Association of Nuclear Medicine (2). Therefore, the inclusion criteria were suspected CADs. In contrast, patients with previously diagnosed CAD, history of myocardial infarction (chest pain or equivalent symptom complex, positive cardiac biomarkers, or typical electrocardiographic changes), percutaneous coronary intervention, or coronary artery bypass grafting were not included *a priori* in our study. All patients were part of an ongoing prospective dedicated database (14). As part of the baseline data collected, clinical teams collected information on traditional cardiovascular risk factors.

Patients were defined as symptomatic if they reported anginal symptoms. Chest pain was classified as non-anginal chest pain, atypical angina, and typical angina, according to the American College of Cardiology/American Heart Association 2002 Guideline Update for Exercise Testing (15). Dyspnea was defined as breathing discomfort that occurs at rest or at lower-than-expected levels of exertion. The body mass index (BMI) of patients was dichotomized with the threshold to 25 kg/m^2^. Patients were considered as having diabetes if they were receiving treatment with oral hypoglycemic drugs or insulin. Hyperlipidemia was defined as a total cholesterol level >6.2 mmol/L or treatment with cholesterol-lowering medication. Hypertension was defined as a blood pressure >140/90 mmHg on three different occasions or use of antihypertensive medication. A family history of premature CAD was defined as a diagnosis of CAD in a first-degree relative prior to or at 55 years of age in men or 65 years in women. Smoking history was defined as prior or current tobacco use. The review committee of our institution approved this study (Ethics Committee, University Federico II, protocol number 110/17), and all patients gave informed consent.

### Myocardial perfusion imaging

Stress-rest ^99m^Tc-sestamibi SPECT cardiac imaging by physical exercise or pharmacologic stress using dipyridamole was performed according to the recommendations of the European Association of Nuclear Medicine (2). Regadenoson agent for the pharmacologic stress test was used only in 54 of 2,031 patients (2.7%) . Imaging was started 30–45 min after tracer injection using a dual-head rotating gamma camera (E.CAM, Siemens Medical Systems) equipped with a low-energy, high-resolution collimator and connected with a dedicated computer system. No attenuation or scatter correction was used. An automated software program (e-soft, 2.5, QPS, Cedars-Sinai Medical Center, Los Angeles, CA) was used to calculate the scores, incorporating both the extent and severity of perfusion defects using standardized segmentation of 17 myocardial regions (16). Perfusion defects were expressed as a summed stress score, representing the total abnormal myocardium. A summed stress score >3 was considered abnormal.

### PET/CT imaging

Rest and stress ^82^Rb cardiac PET/CT cardiac imaging was performed according to the SNMMI/ASNC/SCCT guidelines (5). Scans were acquired using a Biograph mCT 64-slice system (Siemens Healthcare). For both rest and stress images 1,110 MBq of ^82^Rb was injected intravenously with a 7-min list-mode PET acquisition. Dynamic PET acquisition was started at rest, followed by adenosine pharmacologic stress (140 µg kg^−1^ min^−1^ for 4.5 min, with tracer administration between 2 and 2.5 min). Rest and stress dynamic images were reconstructed into 26 time frames (12 × 5 s, 6 × 10 s, 4 × 20 s, and 4 × 40 s; total, 6 min) using the vendor standard ordered subsets expectation maximization 3D reconstruction (2 iterations, 24 subsets) with 6.5-mm Gaussian post-processing filter. In addition, the images were corrected for attenuation using the low-dose CT. The heart rate, systemic blood pressure, and 12-lead ECG were recorded at baseline and throughout the infusion of adenosine. Evaluation of cardiac perfusion defects by PET was obtained with a procedure analogous to those used for SPECT. In our study population, 35 patients had both SPECT and PET MPI. On average, the time interval between the two different MPIs was 2.7 years (SD = 2.2 years).

### Statistics

Continuous variables were expressed as mean ± standard deviation and categorical data as percentages. Differences between groups were analyzed by Student’s *t*-test or *χ*^2^ test, as appropriate. Multivariable logistic regressions were performed considering myocardial perfusion imaging findings as the dependent variable, while independent variables were age, gender, BMI, chest pain symptoms, dyspnea, diabetes, hypertension, hyperlipidemia, family history of CAD, and smoking. As a reference for the BMI, we considered patients with values less than 25 kg/m^2^ normal weight and those above obese. By the multivariable logistic regressions assessed for patients undergoing SPECT or PET, we developed a nomogram for each cohort. The performance of these models was evaluated by receiver operating characteristic (ROC) curves. For each cohort, data were split randomly into two parts: training/test (80%) and validation (20%). For the training/test data, we applied a twofold cross-validation method, repeated twice. The optimal cutoff point for ROC curves was computed by Youden's index, which maximizes the sensitivity and specificity. The 95% confidence intervals (CIs) for the area under ROC curves (AUC) were obtained by 1,000 bootstrap resampling. Two-sided *P*-values <0.05 were considered statistically significant. Statistical analysis was performed using the R software, version 4.3.1 (The R Foundation for Statistical Software, Vienna, Austria).

## Results

The demographic data and clinical characteristics of patients who underwent SPECT or PET cardiac imaging are reported in Table 1.

**Figure 1:**
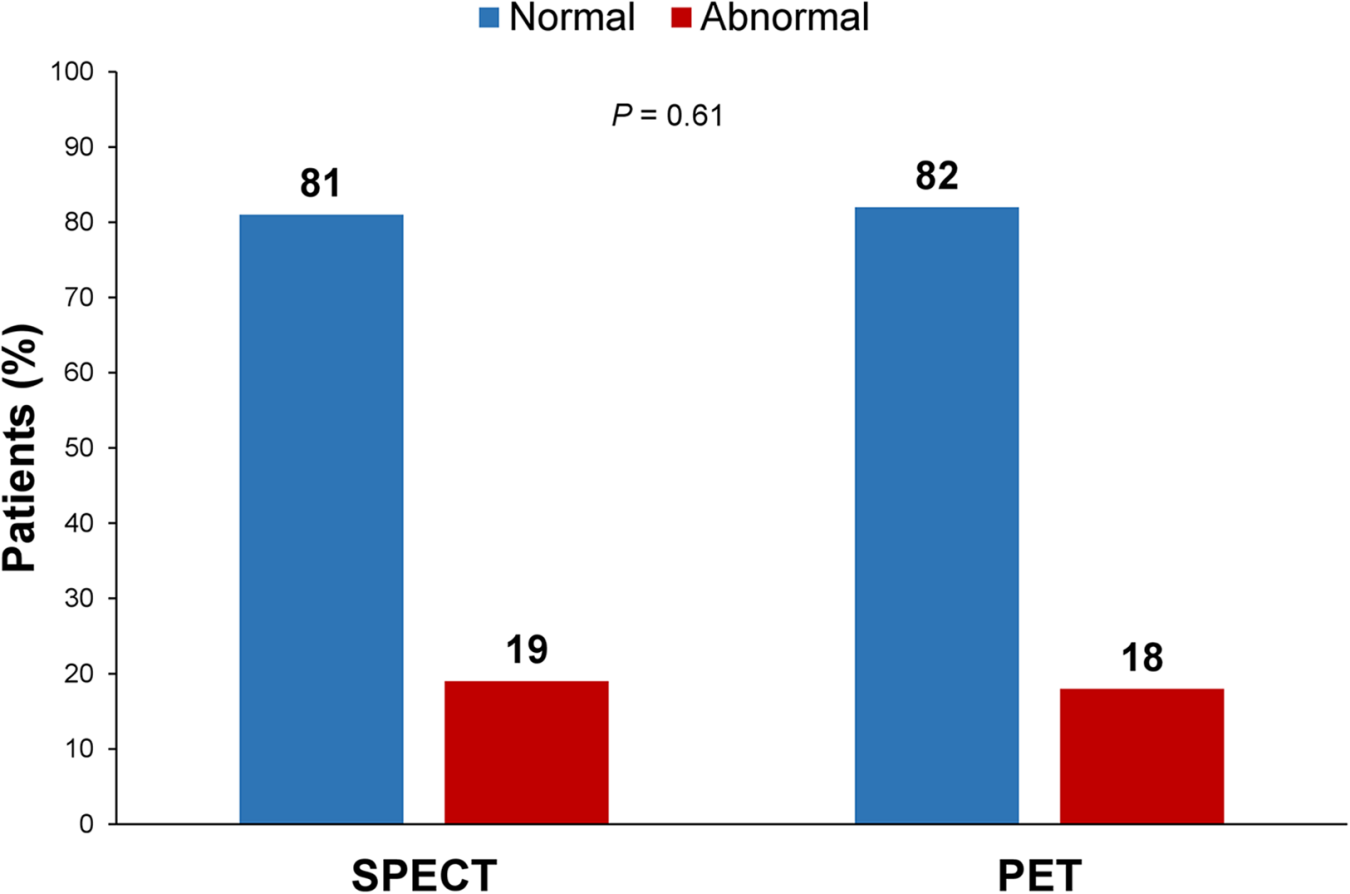
Percentage of patients with normal and abnormal myocardial perfusion findings at SPECT and PET cardiac imaging. The comparison of myocardial perfusion imaging findings between the two cohorts showed no significant difference (*P* = 0.61).

Figure 1 shows the percentage of patients with normal and abnormal myocardial perfusion findings at SPECT and PET cardiac imaging. The prevalence of abnormal myocardial perfusion findings was 19% for SPECT and 18% for PET (*P* = 0.61).

**Table 1:** Characteristics of the study population according to the imaging procedure.

	SPECT (*n* = 4,397)	PET (*n* = 2,457)	*P*-value
Age (years)	63 ± 11	60 ± 13	<0.001
Male gender, *n* (%)	2,675 (61)	1,229 (50)	<0.001
BMI, *n* (%)	3,198 (73)	1,956 (80)	<0.001
Angina, *n* (%)	1,598 (36)	1,108 (45)	<0.001
Dyspnea, *n* (%)	2,020 (46)	678 (27)	<0.001
Diabetes, *n* (%)	1,341 (30)	670 (27)	<0.005
Hyperlipidemia, *n* (%)	2,536 (58)	1,534 (62)	<0.001
Hypertension, *n* (%)	3,500 (80)	1,780 (72)	<0.001
Family history of CAD, *n* (%)	2,162 (49)	1,142 (46)	<0.05
Smoking, *n* (%)	2,086 (47)	779 (32)	<0.001

Table 2 illustrates the characteristics of the study population according to myocardial perfusion findings at SPECT and PET cardiac imaging. A crude statistical significance for both cohorts was observed for age, gender, and diabetes. Moreover, smoking and stress test type were significant for SPECT MPI, while hyperlipidemia and hypertension were significant for PET MPI.

**Table 2:** Characteristics of the study population according to myocardial perfusion findings at SPECT and PET cardiac imaging.

	SPECT (*n* = 4,397)			PET (*n* = 2,457)		
	Normal (*n* = 3,571)	Abnormal (*n* = 826)	*P*-value	Normal (*n* = 2,011)	Abnormal (*n* = 446)	*P*-value
Age (years*)*	62 ± 11	64 ± 11	<0.001	59 ± 13	65 ± 12	<0.001
Male gender, *n* (%)	2,049 (57)	626 (76)	<0.001	939 (47)	290 (65)	<0.001
BMI, *n* (%)	2,600 (73)	598 (72)	0.84	1,599 (80)	357 (80)	0.85
Angina, *n* (%)	1,323 (37)	275 (33)	0.05	920 (46)	188 (42)	0.18
Dyspnea, *n* (%)	1,640 (46)	376 (45)	0.89	535 (27)	139 (31)	0.06
Diabetes, *n* (%)	1,048 (29)	293 (35)	<0.001	499 (25)	171 (38)	<0.001
Hyperlipidemia, *n* (%)	2,082 (58)	474 (57)	0.91	1,224 (61)	310 (70)	<0.001
Hypertension, *n* (%)	2,837 (79)	663 (80)	0.63	1,412 (70)	368 (83)	<0.001
Family history of CAD, *n* (%)	1,771 (50)	391 (47)	0.26	958 (48)	188 (42)	0.04
Smoking, *n* (%)	1,636 (46)	450 (54)	<0.001	633 (31)	146 (33)	0.65
Pharmacological stress, *n* (%)	1,537 (43)	494 (60)	<0.001	2,011 (100)	446 (100)	—

Table 3 shows the results of multivariable logistic regression results in the study population according to the imaging procedure. For patients who underwent SPECT imaging, age, gender, smoking, and stress test type were significant predictors of abnormal findings. Age, gender, diabetes, and hypertension were significant predictors of abnormal findings for patients undergoing PET imaging. The risk of abnormal myocardial perfusion imaging related to age was greater in patients undergoing PET compared to those undergoing SPECT (OR 4% vs. 1% per year). On the other hand, for the male gender OR was higher in the SPECT cohort (2.52 vs. 2.06). In this cohort, smoking increased the risk of abnormal perfusion imaging by 24%, while exercise was a protective factor with respect to the pharmacological stress test (OR 2.12). Among patients undergoing PET imaging, diabetes and hypertension increased the risk by 63% and 37%, respectively.

**Table 3:** Results of multivariable logistic regression results in the study population according to the imaging procedure.

	SPECT (*n* = 4,397)				PET (*n* = 2,457)			
	Estimate	SE	*P*-value	Odds ratio (95% CI)	Estimate	SE	*P*-value	Odds ratio (95% CI)
Intercept	−3.047	0.276	<0.001	—	−4.872	0.365	<0.001	—
Age	0.009	0.004	<0.05	1.01 (1.00–1.02)	0.041	0.005	<0.001	1.04 (1.03–1.05)
Male gender	0.924	0.094	<0.001	2.52 (2.10–3.03)	0.721	0.115	<0.001	2.06 (1.65–2.58)
BMI	−0.052	0.092	0.57	0.95 (0.79–1.14)	0.0004	0.138	0.99	1.00 (0.77–1.32)
Angina	0.033	0.087	0.70	1.03 (0.87–1.23)	0.028	0.113	0.80	1.03 (0.82–1.28)
Dyspnea	0.004	0.083	0.96	1.00 (0.85–1.18)	0.026	0.121	0.83	1.03 (0.81–1.30)
Diabetes	0.107	0.087	0.22	1.11 (0.94–1.32)	0.486	0.117	<0.001	1.63 (1.29–2.05)
Hyperlipidemia	−0.048	0.083	0.57	0.95 (0.81–1.12)	0.08	0.121	0.51	1.08 (0.86–1.38)
Hypertension	−0.105	0.105	0.32	0.90 (0.73–1.11)	0.315	0.143	<0.05	1.37 (1.04–1.82)
Family history of CAD	0.067	0.081	0.41	1.07 (0.91–1.25)	−0.051	0.112	0.65	0.95 (0.76–1.18)
Smoking	0.211	0.081	<0.01	1.24 (1.05–1.45)	0.008	0.118	0.95	1.01 (0.80–1.27)
Pharmacological stress	0.751	0.084	<0.001	2.12 (1.80–2.50)	—	—	—	—

SE, standard error.

**Figure 2:**
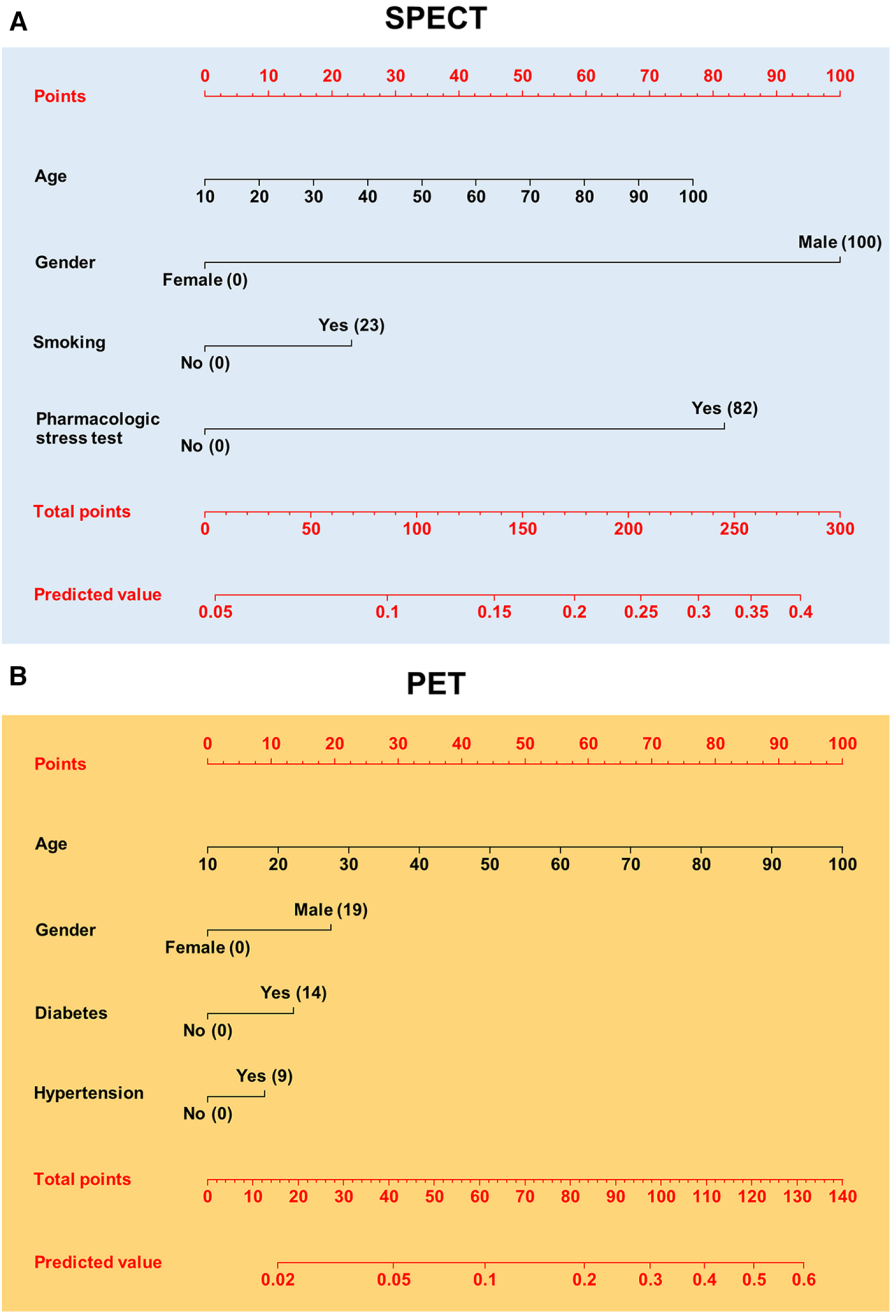
Nomograms for pretest calculation of patients to referral SPECT (**A**) or PET (**B**). The points related to risk factors appear in parentheses. Corresponding to total points, the predicted value for an abnormal MPI is given.

Figure 2 shows the nomograms obtained by significant risk factors at the multivariable logistic regression. For the age continuous covariate, associated points are on a continuous scale. Dichotomous covariates have two possible values, with zero as a reference. To the covariate with greater weight, associated points assume values in the 0–100 interval. By the sum of the points associated with the covariates related to a patient, it is possible to obtain the total points. Corresponding to that, the probability of an abnormal outcome for that patient is available as a predicted value.

**Figure 3:**
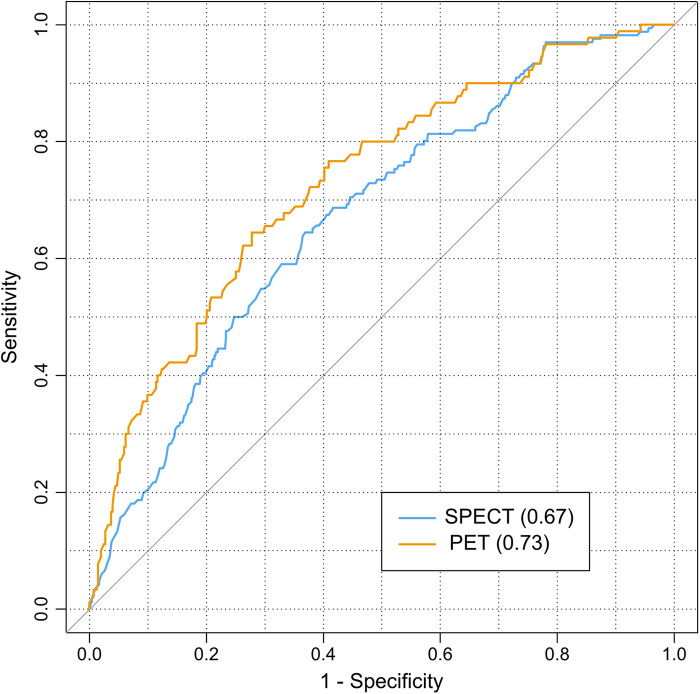
ROC curves related to SPECT and PET logistic models used for nomograms. The area under ROC curves appear in parentheses. The diagonal line represents the no-effect line for models.

Figure 3 reports ROC curves for the two models related to nomograms derived from patients undergoing SPECT or PET. The cutoff point (sensitivity and specificity) for the ROC curve derived by the SPECT model was 0.18 (0.65, 0.63), while for the PET model it was 0.20 (0.64, 0.72). The AUC (95% CI) resulted in 0.67 (0.63–0.71) and 0.73 (0.67–0.78) for SPET and PET MPI, respectively.

## Discussion

Our study shows that the cohorts of patients with suspected CAD undergoing SPECT or PET myocardial perfusion imaging in our academic center from 2012 to 2022 had different clinical characteristics. First of all, patients undergoing PET were younger than those referred to SPECT. Consequentially, this last cohort in general had a significantly higher percentage of subjects with risk factors than the PET cohort did. The different clinical conditions between the cohorts are reflected in the statistical significance of crude variables, evaluated with respect to myocardial perfusion findings. In particular, among risk factors, only age, gender, and diabetes were significant for both cohorts. Moreover, other risk factors only significant for SPECT imaging were not significant for PET and vice versa. To explain these differences, we also have to consider other causes. Basically, referring patients to SPECT or PET/CT is determined by a clinical evaluation, based on patients’ symptoms and previous instrumental investigation. Concerning symptoms, angina could be a motivation for choosing PET/CT due to suspicion of coronary calcium. In this case, the proportion of our patients with angina was 45% and 36%, respectively, for those undergoing SPECT and PET MPI. The correction for attenuation could also determine the choice of a PET examination for both female and obese patients. In our cohorts, we have a proportion of 39% SPECT vs. 50% PET for females, and we have 73% SPECT vs. 80% PET for obese subjects. We cannot exclude that financial reimbursement played a role in each patient's MPI chosen technique. Indeed, the cost of a cardiac PET is very high compared to a cardiac SPECT (1,072 euros vs. 135 euros). This difference may make the choice of SPECT preferable to PET. Besides the financial consideration, it should be noted that our SPECT is prevalently dedicated to cardiac patients, while PET/CT is more frequently used for other patients (in particular, oncological patients). Thus, increased availability of SPECT than PET/CT resulted in a greater number of SPECT examinations. Therefore, this aspect and the lower cost of SPECT might have influenced clinicians to refer to this MPI examination rather than PET/CT when it was not strictly necessary. Other random variables can play a role in decision-making, such as radionuclide and machine availability at short notice. Based on these considerations, we can assume that the two cohorts have a random component. The contribution of variables belonging to only one cohort can also have an important role. In this sense, we verified that the type of stress test determined the loss of significance for diabetes at multivariable logistic regression in patients undergoing SPECT. All these components are reflected in both crude significant variables and significant covariates at multivariable logistic regressions, contributing to the observed results.

However, these results are not surprising, because risk factors depend on the study population. More generally, in epidemiology, geographic, ethnic, and socio-economic conditions are normally considered when similar studies are compared. In any case, demographic characteristics and diabetes are recurrent significant factors in cardiovascular studies carried out in high-income countries.

Despite the differences between the two cohorts, the prevalence of abnormal myocardial perfusion findings was very similar between the two cohorts. This result likely indicates that similar referral criteria were applied for patients undergoing SPECT and PET cardiac imaging.

Through multivariable logistic regression, each variable was adjusted by removing the effect of the other covariates. As expected, some variables lost statistical significance in both cohorts. Age, gender, smoking, and stress test type were significant risk factors in the SPECT imaging cohort. The first three risk factors are recurrent in cardiovascular patients, while the use of a pharmacological stress test generally indicates a worse health condition in patients who were not able to perform the exercise. Beyond age and gender, the SPECT imaging cohort showed a statistical significance for diabetes and hypertension, all of which are traditional cardiovascular risk factors.

Despite the difference observed between cohorts related to the clinical characteristics, age and gender were the common risk factors net of the effect of other covariates. Therefore, these two factors were demonstrated to be independent of the cohorts’ studied characteristics.

Nevertheless, the interpretation of these results should be considered in the following context. On one hand, our study considered only patients with suspected CAD. In patients with known CAD we would have observed a greater number of significant risk factors. On the other hand, cardiovascular risk factors may decline over time. In fact, risk evaluation obtained in historical studies is decreased over time (17–20). Furthermore, a recent study demonstrated the decline of typical angina (21).

The analysis of risk factors has multiple applications in the cardiovascular field. Beyond obtaining epidemiological information, risk evaluation is also used to obtain pretests, which are tools in computing the probability of obtaining an abnormal result in the myocardial perfusion findings test. A pretest is performed before the referral of a patient for myocardial perfusion study, and it aids physicians in decision-making (17–20). For this purpose, we developed a nomogram for each cohort understudy, to use for patients admitted in our academic center. In this way, based on clinical history and symptoms of a patient, physicians can also consult the nomogram in case of doubt before carrying out the test. Further, clinicians who evaluate the state of a patient based on anamnesis, symptoms, and instrumental tests, may have uncertainties about referring him/her for MPI. This examination may be useless without further information than what is already known. In this case, clinicians should evaluate the risks and benefits for the patient. In this way, the nomograms can be utilized to determine the risks associated with the patient and help clinicians in deciding the MPI test. The risk calculation for patients should be evaluated according to MPI type. Despite the apparent differences between the two nomograms, risk calculations are very similar concerning the common covariates, that is, the demographic characteristics. For example, a 60-year-old male without other significant covariates has a risk of approximately 16% for both nomograms. Incremental risk is about 3% for smoking, according to the SPECT nomogram, and 10% for diabetes, according to the PET nomogram. The PET nomogram model had a slightly higher performance than the SPECT, however not significant in comparing the two 95% CI. In relation to what has been discussed, these tools should only be used in populations with characteristics similar to our patients. However, other scholars could validate our models using the data reported in this study. In fact, from models such as those obtained in this study, it is possible to perform external validations by multivariate logistic regression coefficients. The purpose of this technique is to verify whether a model is adaptable to other cohorts or from different countries from where it was obtained (22, 23).

Lastly, studies on cardiovascular risk factors are also performed using machine learning algorithms. In particular, support vector machines, neural networks, naïve Bayesian, boosting, and other procedures are used for clinical evaluations of cardiovascular patients (24–26). In light of these considerations, the study of risk factors with large cohorts, comparing models, and using machine learning algorithms are desirable for advancing cardiology diagnostics and therapeutics.

In this study, we considered patients undergoing MPI from January 2012 to December 2022. We made this choice in order to consider study populations over time and reduce differences among patients due to changes in treatments and lifestyle. In our previous study, we computed the pretest nomogram including 5,601 patients with suspected CAD undergoing a stress SPECT MPI at our academic center between January 2006 and April 2019. In this case, beyond age, male gender and smoking were significant risk factors for angina and diabetes too (6). Our more contemporary study population has excluded some factors such as angina, which seems to be in decline (21). Indeed, comparing the nomograms between the two studies we note a risk reduction quantifiable in approximately 5% for the more recent study population. A generalized decrease of some risk factors for both patients with CAD and suspected of CAD was also observed in another study concerning their temporal trend from 2006 to 2017 (11). Regarding the studies on our patients undergoing PET/CT, in another article, we compared several machine learning pretest approaches to predict stress-induced ischemia (25). In that study, we obtained similar ROC curves for training/test and validation subsets related to the multivariable logistic regression, yet we did not consider any nomogram calculation. In this study, we computed the nomogram for this algorithm that classifies new patients coherently with the training/test phase.

## Conclusions

Our study highlighted that patients with suspected CAD, belonging to two different cohorts—undergoing SPECT or PET stress myocardial perfusion imaging—can have different cardiovascular risk factors, being predictors of an abnormal MPI study. Crude variables, age, gender, and diabetes were significant for both cohorts. Net of the effect of other covariates, age and gender were the only risk factors in common between both cohorts. In addition, smoking and type of stress test were significant for the SPECT cohort; in contrast, diabetes and hypertension were significant for the PET cohort. Nomograms obtained by significant risk factors for the two cohorts can be used by clinicians to evaluate the risk of an abnormal outcome.

## Data Availability

The raw data supporting the conclusions of this article will be made available by the authors, without undue reservation.
